# In-Situ TEM Investigation of Helium Implantation in Ni-SiOC Nanocomposites

**DOI:** 10.3390/ma16041357

**Published:** 2023-02-06

**Authors:** Bingqiang Wei, Wenqian Wu, Jian Wang

**Affiliations:** Department of Mechanical and Materials Engineering, University of Nebraska-Lincoln, Lincoln, NE 68588, USA

**Keywords:** Ni-SiOC nanocomposite, in-situ TEM, He implantation, He bubble

## Abstract

Ni-SiOC nanocomposites maintain crystal-amorphous dual-phase nanostructures after high-temperature annealing at different temperatures (600 °C, 800 °C and 1000 °C), while the feature sizes of crystal Ni and amorphous SiOC increase with the annealing temperature. Corresponding to the dual-phase nanostructures, Ni-SiOC nanocomposites exhibit a high strength and good plastic flow stability. In this study, we conducted a He implantation in Ni-SiOC nanocomposites at 300 °C by in-situ transmission electron microscope (TEM) irradiation test. In-situ TEM irradiation revealed that both crystal Ni and amorphous SiOC maintain stability under He irradiation. The 600 °C annealed sample presents a better He irradiation resistance, as manifested by a smaller He-bubble size and lower density. Both the grain boundary and crystal-amorphous phase boundary act as a sink to absorb He and irradiation-induced defects in the Ni matrix. More importantly, amorphous SiOC ceramic is immune to He irradiation damage, contributing to the He irradiation resistance of Ni alloy.

## 1. Introduction

Strong, ductile, thermally stable and irradiation-tolerant structural materials are in urgent demand to improve the safety and efficiency of advanced nuclear reactors [1,2,3,4]. Nickle (Ni) alloys had been developed for structural applications in Molten Salt Reactors (MSRs) because of their durability and resistance to corrosion and mechanical-property degradation at high temperatures [5,6,7,8,9]. However, there are some challenging issues related to their application in advanced nuclear reactors. The most important one is that Ni alloys are particularly prone to Helium (He) embrittlement [10,11]. Abundant He atoms were produced by transmutation from thermal neutrons in reactors: ^58^Ni + n → ^59^Ni and ^59^Ni + n → ^4^He + ^56^Fe [12,13]. As He has an extremely low solubility in metals, it tends to agglomerate and precipitate into He bubbles in nuclear structural materials. He embrittlement occurs due to the segregation of the He bubble at the grain boundary (GB) [14,15], leading to a dramatic reduction in the ductility and toughness of Ni alloys.

Introducing a high density of interfaces, such as the GB and phase boundary (PB), was proposed to improve irradiation tolerance due to their strong sink effect of absorbing irradiation-induced defects such as He and vacancy, giving rise to a low concentration of He-vacancy clusters in metals and thus retarding the nucleation of He bubbles [16,17,18,19,20,21,22]. The enhanced He irradiation resistance of nanocrystalline (NC) Ni is usually manifested as a small bubble size and low bubble density compared to coarse grain samples [16,23], which was attributed to its large area fraction of GBs. However, NC metals exhibit a low thermal stability [24,25], and obvious grain coarsening usually occurred under thermal and mechanical loading [26,27], degrading the mechanical property. Introducing PBs via alloying with refractory elements (such as Ti) to form fine-scale carbides (such as TiC) or intermetallic precipitates (γ’-Ni3 (Ti, Al)) to trap He is somewhat effective in mitigating He embrittlement at intermediate temperatures [28,29,30,31]. Apart from mitigating He embrittlement, the dispersion of secondary-phase precipitates can effectively strengthen materials at high temperatures if they are thermally stable. However, carbides or intermetallic particles may coarsen or dissolve at high temperatures (up to 700 °C and beyond) and under ion irradiation [32,33,34,35,36], and the ability to manage the distribution of He atoms and retain a high temperature strength is thus significantly lost. Therefore, it is urgent to introduce or form high density, thermally stable, fine-scale, dispersed particles in Ni alloys.

In contrast to crystal solids, amorphous SiOC ceramic exhibits a superior irradiation resistance and thermo-mechanical properties (strength and hardness, creep and oxidation) as well as a structural stability at elevated temperatures (>1300 °C) and irradiation [37,38,39,40,41,42]. Recently, Ni-SiOC nanocomposites were prepared by co-sputtering technique, which were composed of NC Ni and an amorphous SiOC phase with an adjustable distribution and feature size [43,44]. In-situ micropillar compression tests indicated that the Ni-SiOC nanocomposites showed a high strength at both room temperature and high temperature, good thermal stability and large plasticity [43,44]. The amorphous SiOC ceramic co-deformed with NC Ni, contributing to a superb plastic flow stability. However, the irradiation behavior of Ni-SiOC nanocomposites is still unknown. The introduction of amorphous SiOC ceramic is supposed to contribute to the irradiation resistance of Ni alloy due to the high thermal stability and irradiation resistance of amorphous SiOC itself and the formation of crystal–amorphous interfaces. To verify this hypothesis, we instigated the irradiation behavior of Ni-SiOC nanocomposites under He implantation associated with in-situ transmission electron microscope (TEM) characterization. The initial microstructures of Ni-SiOC nanocomposites, with different feature sizes and distributions of crystal Ni and amorphous SiOC phase, were used in this study. In-situ TEM irradiation revealed that Ni-SiOC nanocomposites showed a high He irradiation resistance. Both crystal Ni and amorphous SiOC remained stable under He irradiation at 300 °C. The amorphous SiOC phase was immune to He-induced damage because no He bubble was produced inside. Both GB and PB act as a strong sink to absorb He and irradiation-induced defects from Ni. This work provides a strategy to improve the He irradiation resistance of Ni alloy by introducing an amorphous SiOC ceramic phase.

## 2. Experimental Method

**Figure 1:**
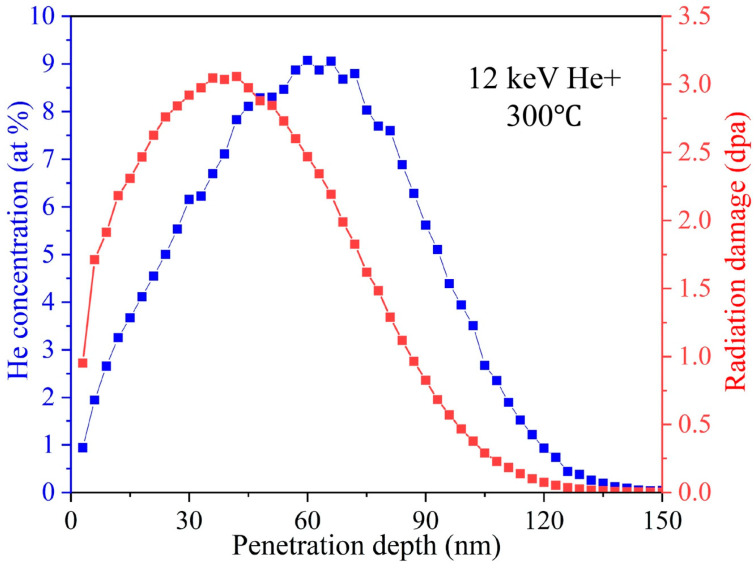
SRIM simulation of He concentration and corresponding displacement damage profiles.

Ni-SiOC nanocomposites with a nominal composition of 75 at.% Ni were prepared by co-sputtering technique using Ni (99.995%), SiC (99.5%) and SiO_2_ (99.995%) targets. More details for the sample synthesis and chemical composition analysis were presented in our previous work [43]. To further tune the microstructure and investigate irradiation behavior with respect to different initial microstructures, the as-deposited sample was then annealed at 600 °C, 800 °C and 1000 °C under vacuum for 1 h, respectively. The annealing-induced microstructure evolution was investigated by performing post-mortem TEM characterization after annealing. TEM samples for in-situ irradiation were prepared by focused ion beam (FIB) lift-out technique using an FEI Helios nanolab 660 dual-beam system, in which the sample was welded on the Cu grid by Pt deposition. The thickness of TEM samples was maintained at ~100 nm. In-situ TEM irradiations were conducted in the Intermediate Voltage Electron Microscope (IVEM) at Argonne National Laboratory using a Hitachi-900 TEM attached with an ion accelerator. He ions with 12 KeV were implanted into the sample with a fluence of 6.6 × 10^16^ ions/cm^2^ at 300 °C, corresponding to a He concentration of up to 9 at.% and an irradiation damage of 3 displacement-per-atom (dpa). The chosen irradiation temperature was balanced by promoting the appearance of He bubbles via improving the defect mobility, while mitigating the softening of the TEM Cu support grid and surface pollution from deposited Pt by diffusion. A GATAN OneView digital camera was used to capture the microstructure evolution during radiation with a speed of 5 frames per second. The depth-dependent He implantation profile and corresponding displacement damage (Figure 1) were simulated by using Stopping and Range of Ions in Matter (SRIM)-2013 software [45], with the ion distribution and quick calculation of damage option using 40, 15, 28 and 28 eV for Ni, Si, O and C [46]. Due to the limit resolution of TEM for in-situ irradiation, only the high-temperature-annealed Ni-SiOC samples underwent an in-situ irradiation investigation.

## 3. Results and Discussion

**Figure 2:**
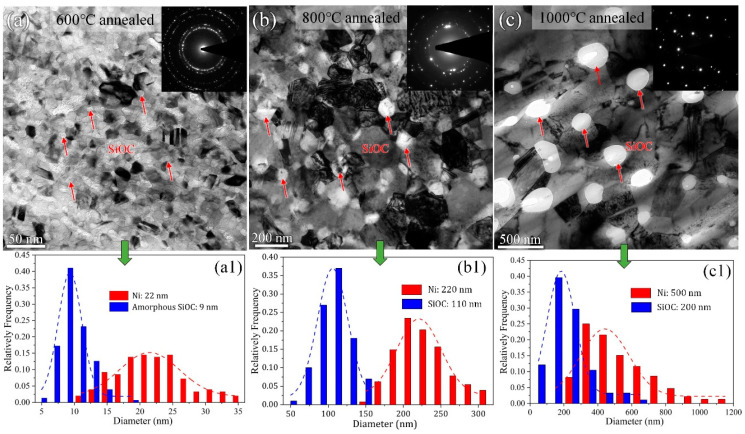
TEM bright field images and corresponding feature size of Ni and SiOC phase for Ni-SiOC nanocomposites: (**a**) 600 °C annealed, (**b**) 800 °C annealed and (**c**) 1000 °C annealed. Amorphous phase is indicated by the red arrows. (**a1**–**c1**) the statistical analysis of characteristic dimensions of Ni and amorphous SiOC phases.

Figure 2 shows the initial TEM microstructure of annealed Ni-SiOC nanocomposites and the corresponding feature size distribution before He implantation. Compared to the core-shell structure with amorphous ceramic SiOC along GBs of NC Ni (~13 nm) in the as-deposited sample [43], both the Ni and amorphous SiOC phase grew with an increasing annealing temperature (Figure 2a–c). The feature and distribution of the SiOC phase is easily distinguishable from the bright contrast due to its amorphous structure, which exhibits no diffraction contrast. As can be seen, the amorphous ceramic SiOC maintains a stable structure, even when annealed at 1000 °C, and the sample maintains a crystal-amorphous dual-phase structure despite the increased feature size. The feature size of Ni and amorphous SiOC is about 22 nm (Ni) and 9 nm (SiOC) in the 600 °C annealed sample (Figure 2(a1)), 220 nm (Ni) and 110 nm (SiOC) in the 800 °C annealed sample (Figure 2(b1)), and 500 nm (Ni) and 200 nm (SiOC) in the 1000 °C annealed sample (Figure 2(c1)). The size variation of crystal Ni is also reflected in the corresponding electron diffraction patterns (insets in Figure 2a–c). Compared to the quick and extensive grain coarsening of NC Ni during high-temperature annealing [47,48], the sub-micron-sized Ni after 1000 °C annealing indicates the relatively high thermal stability of the Ni-SiOC nanocomposite. This should be ascribed to the introduction of the amorphous SiOC ceramic, which has a pinning effect on GB, inhibiting grain growth. As revealed in our previous work [44], except for the amorphous SiOC nanoparticles, the amorphous SiOC phase remained along GBs of NC Ni in the 600 °C annealed sample, indicating that only one type of interface, i.e., crystal-amorphous PB, dominated in the 600 °C annealed sample. Due to the phase separation and obviously increased feature size of Ni and SiOC in the 800 °C and 1000 °C annealed samples, the amorphous SiOC phase was mainly distributed at the triple junction of sub-micron-sized Ni grains, and most GBs were clean without SiOC phase decoration. This indicates that two types of interfaces dominated, i.e., PB and GB, in the 800 °C and 1000 °C annealed samples. Due to the nano-scaled feature size of Ni and SiOC in the 600 °C annealed sample, the area fraction of PBs should be higher than that in the other two samples.

**Figure 3:**
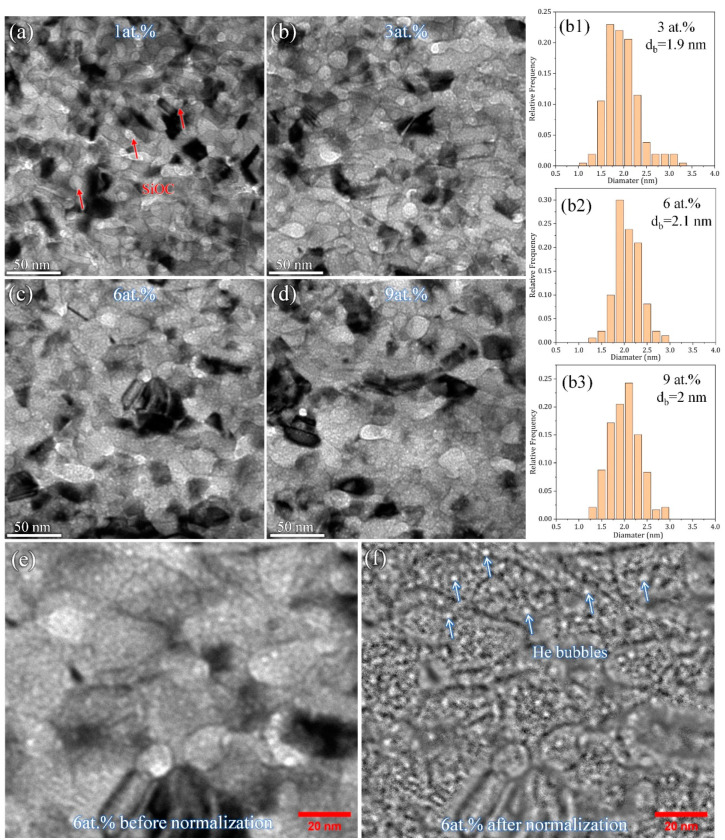
(**a**–**d**) In-situ TEM snapshot images and corresponding distribution of He-bubble size during He implantation for 600 °C annealed sample; the red arrows indicate amorphous phase. One example of a local area image (**e**) before and (**f**) after normalization processing in 600 °C annealed sample. The white arrows indicate He bubbles. (**b1**–**b3**) the statistical analysis of the size of He bubbles.

The in-situ irradiation test was recorded in four parts based on the concentration of implanted He (1 at.%, 3 at.%, 6 at.% and 9 at.%). Figure 3 present the under-focused TEM snapshot images during irradiation and corresponding distribution of the He-bubble size for the 600 °C annealed sample, revealing several important features: (1) few He bubbles formed after 1 at.% He implantation (Figure 3a); (2) no obvious growth of He bubbles (~2 nm) was observed from 3 at.% to 9 at.% He implantation, as shown in Figure 3b–d; (3) the amorphous SiOC phase remained stable, and no obvious grain coarsening of NC Ni occurred during irradiation. The above features indicate a very good thermal stability and He irradiation resistance of the 600 °C annealed Ni-SiOC nanocomposite. Note that the inevitable overlapping between NC Ni and amorphous SiOC nanoparticles resulting from their fine size makes it difficult to identify and track the distribution and evolution of He bubbles. To reduce the influence of bright contrast from the amorphous SiOC phase on the statistical analysis of He bubbles, the TEM image was processed by ImageJ program using the Normalize Local Contrast method, as shown in Figure 3e,f (one example before and after normalization). Previous studies by Su et at. [37,49,50] indicated that amorphous SiOC ceramic is immune to He-induced damage. In contrast to the formation of He bubbles in metals, He in SiOC remains in solution and outgasses from the material via atomic-scale diffusion without damaging the free surface [37,49]. Correspondingly, He bubbles in the 600 °C annealed Ni-SiOC nanocomposite should originate from the NC Ni matrix, and the high density of SiOC nanoparticles and PBs contribute to He irradiation resistance.

**Figure 4:**
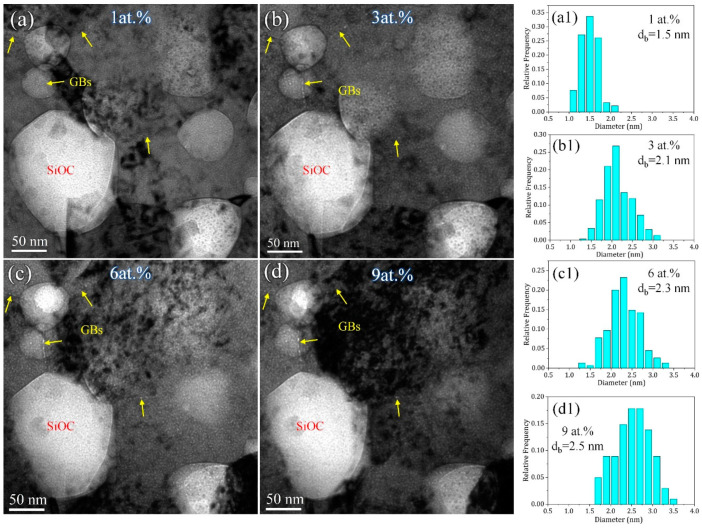
(**a**–**d**) In-situ TEM snapshot images and corresponding distribution of He-bubble size during He implantation for 800 °C annealed sample. (**a1**–**d1**) the statistical analysis of the size of He bubbles.

**Figure 5:**
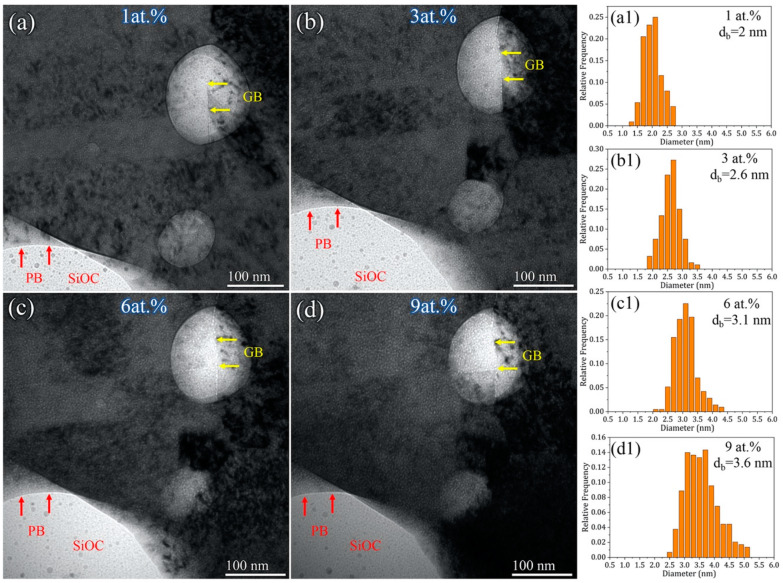
(**a**–**d**) In-situ TEM snapshot images and corresponding distribution of He-bubble size during He implantation for 1000 °C annealed sample. (**a1**–**d1**) the statistical analysis of the size of He bubbles.

Figure 4 presents under-focused TEM snapshot images with an increasing concentration of He implantation and the corresponding distribution of the He-bubble size for the 800 °C annealed sample. Note that most of the SiOC particles are still overlapped with the Ni matrix and GB, which can be identified from the existence of He bubbles on the SiOC phase. The bubble size in the Ni matrix increased with the He concentration, which increased from 1.5 nm at 1 at.% He to 2.5 nm at 9 at.% He (Figure 4(a1–c1)). The accumulation of He bubbles at GBs was observed, as marked by yellow arrows, suggesting that GBs can act as an effective He sink. In addition, no obvious GB migration occurred during in-situ He irradiation, which proves the good thermal stability of the 800 °C annealed Ni-SiOC nanocomposite under He irradiation. The larger feature size of crystal Ni and amorphous SiOC in the 1000 °C annealed sample provided more chances to select a domain without overlapping between them. Figure 5 presents under-focused TEM snapshot images with an increasing concentration of He implantation and the corresponding distribution of the He-bubble size for the 1000 °C annealed sample. Two types of interfaces are marked in Figure 5, GB (yellow arrow) and PB (red arrow). An obvious He-bubble accumulation was observed at GB, while no He accumulation occurred at the crystal-amorphous PB, where the amorphous SiOC particle is supposed to be exposed to the sample surface, according to the Fresnel contrast of PB under under-focused imaging conditions. It is noted that no He bubble was produced in the amorphous SiOC phase, which is consistent with previous work according to which amorphous SiOC ceramic is immune to He-induced damage [37,49]. Statistical analysis of the He-bubble size in the Ni matrix reveals that the bubble size increased with He implantation, from 2 nm at 1 at.% to 3.6 nm at 9 at.% He (Figure 5(a1–c1)). Similar to the 800 °C annealed sample, no obvious GB and PB migrations were observed. Additionally, no large-sized voids formed in either the 800 °C and 1000 °C annealed samples, implying a good He irradiation resistance.

**Figure 6:**
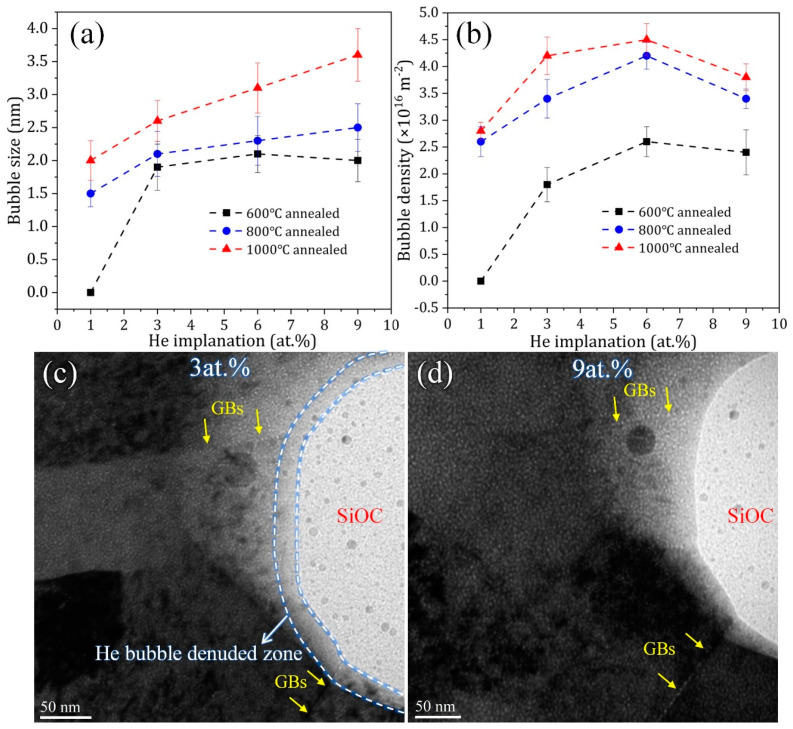
Comparison of (**a**) He-bubble size and (**b**) area density in Ni matrix with increasing implanted He concentration for 600 °C, 800 °C and 1000 °C annealed Ni-SiOC nanocomposites; (**c**,**d**) crystal-amorphous interface with He-bubble-denuded zone and He-bubble accumulation at GB in 1000 °C annealed sample. The arrows indicate the location of grain boundaries.

These in-situ TEM irradiation tests revealed that the Ni-SiOC nanocomposites with different feature sizes of Ni and amorphous SiOC phase remained stable under He irradiation at 300 °C. No grain coarsening of Ni and structure variation of SiOC occurred. He bubbles are only observed in the Ni matrix and tend to accumulate at Ni GB. The amorphous SiOC is immune to He irradiation, and no He-bubble segregation occurred at crystal-amorphous PBs. In addition, both GB and PB are stable during irradiation. Figure 6a,b reveal the He-bubble size and area density in the Ni matrix with an increasing implanted He concentration for Ni-SiOC nanocomposites with different statuses, respectively. Since it is difficult to separate NC Ni and amorphous SiOC particles in the 600 °C annealed sample, the area of the Ni matrix is taken according to its area fraction, which is about 50%. For the measurement of the area density of the He bubble, 3–5 local regions were collected, and the average value with the standard error was used. The He-bubble size and density in the 600 °C annealed sample are both the smallest/lowest, followed by the 800 °C annealed sample and then the 1000 °C annealed sample. A small and low density of bubbles implies less bubble-induced swelling and thus a better He irradiation tolerance. This should be mainly attributed to the high density of amorphous SiOC nanoparticles and the high area fraction of the crystal-amorphous interface in the 600 °C annealed sample. Firstly, amorphous SiOC ceramic was proved to be immune to He-induced damage; He outgasses via the diffusion of isolated He atoms without surface damage and thus with no formation of He bubbles or precipitation [37,49]. This is related to the relatively low interstitial-formation energy, migration energy and dimer-interaction energy of He in amorphous SiOC ceramic, as revealed by density functional theory (DFT) calculations [49]. Lower interstitial-formation and migration energies imply less driving force for He to precipitate and a high He diffusion rate. Dimer-interaction energy determines the tendency of He to cluster into precipitate nuclei. Secondly, the high density of crystal-amorphous PBs in the 600 °C annealed sample can act as a defect sink to absorb He and other irradiation-induced defects from Ni, contributing to the enhanced irradiation resistance. With an increasing annealing temperature, the crystal Ni and amorphous SiOC phase tend to separate, which is associated with their obviously increased feature size. Correspondingly, the number density of amorphous SiOC particles and area fraction of crystal-amorphous PBs in unit area in the 800 °C and 1000 °C annealed samples should be lower, producing a relatively larger He-bubble size and density. However, the existence of large-sized amorphous SiOC particles and GBs is still beneficial for He irradiation resistance. Note that He bubbles show a reduced density at 9 at.% for these Ni-SiOC nanocomposites (Figure 6b), which might be related to the outgassing of He bubbles from the amorphous SiOC phase and to migration and coalescence, considering that the irradiation temperature is 300 °C. Figure 6c–d show another local area of the 1000 °C annealed sample during He irradiation, in which the amorphous SiOC is exposed to the sample surface. Clearly, no He bubble existed in the amorphous SiOC phase. More importantly, a He-bubble-denuded zone (marked by a white dashed line in Figure 6c) was observed close to the amorphous particle. This supports our statement that crystal-amorphous interfaces act as a defect sink to absorb He bubbles and defects from crystal Ni and that He bubbles can be directly outgassed from the SiOC phase, producing a poor He zone at the crystal-amorphous interface. In addition, GBs with obvious He-bubble accumulation were connected to amorphous SiOC particles (marked by yellow arrows in Figure 6d), which could act as a channel to transport He to the amorphous SiOC phase, reducing the He density in the Ni matrix.

## 4. Conclusions

Ni-SiOC nanocomposites were prepared by co-sputtering technique followed by high-temperature annealing (600 °C, 800 °C and 1000 °C). Compared to the sub-micron-sized crystal Ni and amorphous SiOC in the 800 °C and 1000 °C annealed samples, the 600 °C annealed sample exhibited a nano-scaled feature size for crystal Ni and amorphous SiOC. In-situ TEM He implantation tests revealed that the Ni-SiOC nanocomposites exhibited a good He irradiation resistance, which was summarized by the following aspects: (1) amorphous SiOC ceramic remained stable during irradiation, and no obvious grain coarsening of Ni existed; (2) amorphous SiOC ceramic was immune to He irradiation, as proved by there being no He bubbles inside; (3) both GB and PB can act as a defect sink to absorb He, and no accumulation of He bubbles existed at PB, since He bubbles can outgas from the SiOC phase without surface damage; (4) no migration of GB and PB occurred during irradiation, and no large-sized void formed. The 600 °C annealed sample showed a better He irradiation resistance, as manifested by a smaller He-bubble size and lower density compared with that in the other two samples, which was attributed to the high density of SiOC nanoparticles and high area fraction of crystal-amorphous PBs. This work provides a strategy to improve the He irradiation resistance of Ni alloy by introducing an amorphous SiOC ceramic phase.
